# Peer Review of “Estimating Variance of Log Standardized Incidence Ratios Assessing Health Care Providers’ Performance: Comparative Analysis Using Bayesian, Bootstrap, and Delta Method Approaches”

**DOI:** 10.2196/83798

**Published:** 2025-10-09

**Authors:** Emmanuel Oluwagbade

**Affiliations:** 1 Vanderbilt University, Nashville, TN, United States

**Keywords:** standardized incidence ratio, SIR, performance, health care providers, machine learning, equity

*This is a peer-review report for “Estimating Variance of Log Standardized Incidence Ratios Assessing Health Care Providers’ Performance: Comparative Analysis Using Bayesian, Bootstrap, and Delta Method Approaches.”*

## Round 1 Review

### General Comments

This paper [1] compares 3 approaches for estimating the variance of the log standardized incidence ratio when profiling kidney replacement therapy centers in Australia: (1) the analytical delta method, (2) the nonparametric bootstrap method (5000 resamples), and (3) Bayesian Markov chain Monte Carlo (25,500 iterations, 3 chains). Using 2005‐2023 patient-level data from the Australia and New Zealand Dialysis and Transplant Registry and a random-effects logistic model, the authors evaluated bias, variance, and mean squared error (MSE) and visualized performance via funnel plots. Results indicated similar bias across methods but substantially lower variance and MSE for the Markov chain Monte Carlo method (bias≈0.019; variance=0.00005; MSE=0.00042) compared with the bootstrap method (variance=0.00027; MSE=0.00094).

The topic is practical and timely, yet the manuscript needs clearer model specification, interval coverage evaluation, and streamlined writing before it reaches publishable quality.

### Specific Comments

#### Major Comments

1. There are some concerns around model clarity (Poisson versus logistic language mixed; appendix is missing). Provide complete model equations, a covariate list, and software/code links and justify using the Bernoulli model for a ratio outcome.
2. Interval coverage and type I error absent. Action: add a simulation or internal bootstrap to report 95% interval coverage and false-positive rates for each method.
3. Missing data handling unexplained.Action: quantify missingness, describe any imputation, and list all risk-adjustment covariates.

#### Minor Comments

In Table 1, add units and align decimals.
